# Physicochemical Properties of Different Sulfated Polysaccharide Components from *Laetiporus sulphureus* and Their Anti-Proliferative Effects on MDA-MB-231 Breast Cancer Cells

**DOI:** 10.3390/jof10070457

**Published:** 2024-06-28

**Authors:** Chia-I Jen, Lean-Teik Ng

**Affiliations:** Department of Agricultural Chemistry, National Taiwan University, No. 1, Sec. 4, Roosevelt Road, Taipei 10617, Taiwan; shelly810229@gmail.com

**Keywords:** *Laetiporus sulphureus*, sulfated polysaccharides, physicochemical properties, triple-negative breast cancer, cell cycle arrest, protein microarray

## Abstract

*Laetiporus sulphureus* is an edible and medicinal mushroom widely used in folk medicine for treating cancer and gastric diseases. This study aimed to investigate the physicochemical properties of different sulfated polysaccharide (SPS) components (F1, F2, and F3) isolated from *L. sulphureus* and evaluate their activity against MDA-MB-231 breast cancer cell proliferation. Compared with F1 and F3, the results showed that F2 exhibited the most potent anti-proliferative activity on MDA-MB-231 cells, which could be attributed to the sulfate and protein contents, molecular weight, and monosaccharide composition. F2 inhibited breast cancer cell proliferation by blocking the cell cycle at the G0/G1 phase but not triggering cell apoptosis. In addition, F2 also showed selective cytotoxicity on breast cancer cells. It modulated the expression of proteins involved in G0/G1 phase progression, cell cycle checkpoints, DNA replication, and the TGFβ signaling pathway in MDA-MB-231 cells. This study demonstrated that F2, the medium-molecular-weight SPS component of *L. sulphureus*, possessed the most potent inhibitory effect on MDA-MB-231 cell proliferation by arresting the cell cycle at the G0/G1 phase. The main factors contributing to the differences in the potency of anti-breast cancer activity between F1, F2, and F3 could be the sulfate and protein contents, molecular weight, and monosaccharide composition of SPS.

## 1. Introduction

Recently, mushroom polysaccharides have received significant attention in the medical and functional food industries. Many studies have shown that mushroom polysaccharides possess diverse bioactivities, such as anti-inflammatory, anti-tumor, immunomodulatory, anti-obesity, and hypoglycemic effects [1,2]. The bioactivities of mushroom polysaccharides are significantly affected by their physicochemical properties, including chemical composition, molecular weight, monosaccharide composition, structure, linkage type, etc. [3,4]. These polysaccharides may be heterogeneous, have more than two different molecular weight populations, and possess distinct bioactivities. Studies have shown that two non-digestible polysaccharide components of *Grifola frondosa* had very different chemical structures, and the high-molecular-weight fraction was the major bioactive component [5]. In another study, two fractions of soluble polysaccharides from *Xylaria nigripes* exhibited synergistic anti-inflammatory effects; one was a high-molecular-weight glucan and the other was a low-molecular-weight heteropolysaccharide [6]. Therefore, understanding the physicochemical properties of each component of polysaccharides is a critical way to assess their biological functions and application values.

Sulfated polysaccharides are negatively charged polysaccharides containing hemi-ester sulfate groups on the monosaccharide units. Natural sulfated polysaccharides are mainly derived from seaweeds. Apart from *L. sulphureus*, sulfated polysaccharides have not been reported in mushroom fruiting bodies. These macromolecules have generated much attention in recent years due to their promising pharmacological activities, including anti-coagulant, anti-cancer, anti-inflammatory, anti-viral, and neuroprotective properties [3,7,8]. Several studies have demonstrated that sulfated polysaccharides exhibit better pharmacological properties than non-sulfated polysaccharides [3,9,10]. A positive correlation was noted between the degree of sulfation (DS) and the potency of the anti-coagulant activity of polysaccharides [11]. However, studies also showed that there might be an optimum range of the DS on SPS for immune cell activation [12]. To enhance the inherent bioactivities of mushroom polysaccharides, many researchers used artificial chemical approaches to produce synthetic mushroom-derived sulfated polysaccharides, for example, the chemical modification and sulfate-feeding liquid culture of *Pleurotus ostreatus* [13] and *Antrodia cinnamomea* [14]. However, information on the chemical and pharmacological properties of natural sulfated polysaccharides from mushroom fruiting bodies remains limited.

According to the report of the American Cancer Society and the National Cancer Institute, breast cancer was the most prevalent cancer among females in 2022 [15]. Although triple-negative breast cancer (TNBC) only accounts for 15–20% of all breast cancers, it is a very tricky and aggressive subtype of breast cancer. TNBC is characterized by the lack of expressions of three receptors commonly found in other breast cancer subtypes: estrogen receptor, progesterone receptor, and human epidermal growth factor receptor 2. Patients with TNBC may have a shorter overall survival than those with non-TNBC breast cancer due to its highly metastatic property and drug-resistant characteristics. Many researchers have been exploring natural products for new drugs that can specifically destroy cancer cells but are less harmful to the human body. For centuries, mushroom polysaccharides have been used in folk medicines to treat cancer in Asia. Medicinal mushrooms, such as *Coriolus versicolor*, *Ganoderma lucidum*, *G. frondosa*, and *Lentinus edodes*, are reported to have promising anti-breast cancer activity and low toxicity [16,17]. In addition, sulfated polysaccharides extracted from marine algae, such as *Porphyridium sordidum*, *Fucus vesiculosus*, and *Sargassum wightii*, have been shown to exhibit good cytotoxic effects on human TNBC cell lines [18,19,20]. These studies indicate that mushroom polysaccharides and sulfated polysaccharides are potential drug candidates and can be used as adjuvants for breast cancer therapy.

*Laetiporus sulphureus* (Bull.) Murrill., also known as “chicken of the woods,” is a wood decay basidiomycete with yellow-orange basidiocarps, and it is a common edible and medicinal mushroom in Asia, Europe, and North America. This fungus has been widely used as a folk medicine for treating different types of cancers and gastric diseases, and was reported to exhibit anti-cancer, anti-inflammatory, anti-bacterial, and hypoglycemic properties [21,22]. Various unique bioactive secondary metabolites, such as polysaccharides, lectin, laetiporic acids, triterpenoids, and especially the sulfated polysaccharides (SPS), were found in *L. sulphureus* fruiting bodies [10,23]. In our previous study, SPS isolated from *L. sulphureus* fruiting bodies was shown to possess potent anti-breast cancer activity [24]. According to the results of molecular weight analysis, SPS contained three different molecular weight components. However, their physicochemical properties and anti-breast cancer activity remain unclear. Therefore, this study investigated the physicochemical properties of different *L. sulphureus* SPS components and their activity against MDA-MB-231 cell proliferation, and the mechanisms of action of the most potent SPS component were examined by protein microarray. This study was the first to demonstrate the differences in physicochemical properties between various SPS components (F1, F2, and F3) of *L. sulphureus* fruiting bodies and their anti-proliferative effects on MDA-MB-231 breast cancer cells and clarify the detailed mechanisms of action of the most potent SPS component.

## 2. Materials and Methods

### 2.1. Materials

Dried powder of *L. sulphureus* fruiting bodies was obtained from Kang Jian Biotech Co., Ltd. (Nantou, Taiwan). The specimen was deposited at GenBank with the accession number MT269336.

Dextrans (5, 25, 150, 270, 670, 1100, and 1400 kDa), monosaccharide standards (D-galactose, D-glucose, D-mannose, L-rhamnose, D-ribose, D-galacturonic acid, D-glucuronic acid, L-arabinose, D-xylose, and L-fucose), methylthiazolyldiphenyl-tetrazolium bromide (MTT), fetal bovine serum (FBS), protease inhibitor, and phosphatase inhibitor were purchased from Sigma-Aldrich (St. Louis, MO, USA). Papain (EC 3.4.22.2) and cisplatin were purchased from Acros Organics (Geel, Belgium). Dulbecco’s modified Eagle’s Medium (DMEM), minimum essential medium α (MEM-α), 0.25% trypsin-EDTA, penicillin, and streptomycin were purchased from Cytiva (Marlborough, MA, USA). RNase A and propidium iodide were obtained from Bioman Scientific Co., Ltd. (New Taipei City, Taiwan). RIPA lysis buffer was purchased from Visual Protein (Taipei, Taiwan). All other chemicals used in this study were of analytical grade.

### 2.2. Extraction of Sulfated Polysaccharides (SPS)

SPS was prepared according to the method described previously [25]. Briefly, 1 g of *L. sulphureus* powder was mixed with 40 mL of extraction buffer containing 0.1 M acetate buffer (pH 5.5), 5 mM cysteine, 100 mg papain, and 5 mM EDTA. After a 24 h extraction at 60 °C, the mixture was centrifuged at 2000× *g* for 10 min, followed by collecting the supernatant. The precipitate was mixed with another 40 mL extraction buffer and then subjected to a second time of 24 h extraction under the same condition. The supernatants from two separate extractions were combined, followed by adding the 3.75-fold volume of 95% ethanol and kept at 4 °C overnight. After centrifuging at 9000× *g* for 10 min at 4 °C, the precipitate was collected and redissolved with 100 mL of deionized water. The sample solution was transferred to a dialysis tubing (MW cut-off: 12–14 kDa; Spectrum Laboratories Inc., Rancho Dominguez, CA, USA) and dialyzed against deionized water for 72 h at 4 °C with the deionized water refreshed daily. The supernatant was collected by centrifuging at 9000× *g* for 10 min at 4 °C, followed by lyophilization to obtain the sulfated polysaccharides named SPS.

### 2.3. Preparation of F1, F2, and F3 from SPS

An amount of 2 grams of SPS was taken and dissolved in 1000 mL of deionized water. The separation of F1, F2, and F3 from SPS was performed using a tangential flow filtration system coupled with different molecular weight cutoff (MWCO) modified polyethersulfone hollow fiber columns (Spectrum Laboratories Inc.). The retentate was collected after 48 h of separation with a 100 kDa MWCO column and then lyophilized to obtain the high-molecular-weight SPS component named F1. The permeate of a 100 kDa MWCO column was then subjected to a 10 kDa MWCO column for another 48 h of separation. The retentate obtained was the medium-molecular-weight SPS component named F2, and the filtrate was the low-molecular-weight SPS component named F3.

### 2.4. Analysis of Carbohydrate and Protein Contents

The content of carbohydrates was measured by the phenol sulfuric acid method, and glucose (0, 20, 40, 60, 80, and 100 μg/mL) was used as a standard [26]. The protein content was determined by Bradford’s assay using bovine serum albumin (0, 20, 40, 60, 80, and 100 μg/mL) as a standard [27].

### 2.5. Estimation of Uronic Acid Content

The uronic acid content was determined by Blumenkrantz and Asboe-Hansen’s method [28]. In brief, 0.2 mL of sample solution (100 μg/mL, dissolved with deionized water) was added to 1.2 mL of borate–sulfuric acid reagent (12.5 mM sodium tetraborate in sulfuric acid), followed by incubating at 100 °C for 5 min. After cooling to room temperature, 20 μL of 0.15% 3-phenylphenol (dissolved in 0.5% NaOH) was added, and the absorbance at 520 nm was measured using a microplate reader (Infinite M200 PRO, Tecan Group Ltd., Männedorf, Switzerland). Glucuronic acid (0, 20, 40, 60, 80, and 100 μg/mL) was used as a standard.

### 2.6. Determination of Sulfate Content

The sulfate content was measured by the barium chloride–gelatin method [9]. Briefly, 2 mg of the sample was acid-hydrolyzed with 2 N trifluoroacetic acid (TFA) at 80 °C for 6 h, then TFA was removed by a rotary evaporator. The residue was redissolved with 0.5 mL of deionized water, and 0.3 mL was taken to mix with 0.7 mL of barium chloride–gelatin reagent, followed by incubation at room temperature for 10 min. The absorbance at 360 nm was measured using a microplate reader (Infinite M200 PRO). MgSO_4_ was used as a standard. The barium chloride–gelatin reagent was prepared by dissolving 0.5 g of gelatin in 100 mL of deionized water at 60 °C and incubated at 4 °C overnight. After mixing with 0.5 g of BaCl_2_ and 1.25 mL of 12 N HCl, the reagent was stored at 4 °C for further use.

### 2.7. Analysis of Homogeneity and Molecular Weight

Molecular weights of F1, F2, and F3 were analyzed using a high-performance gel-filtration chromatography system coupled with a refractive index detector (HPGFC-RI) and a TSKgel GMPWXL column (300 × 7.8 mm, 13 μm; Tosoh, Tokyo, Japan) [5]. The mobile phase was 50 mM NaNO_3_ solution containing 0.05% NaN_3_, and the flow rate was set at 0.6 mL/min. Dextrans (5, 25, 150, 270, 670, 1100, and 1400 kDa) were used as reference standards.

### 2.8. Determination of Monosaccharide Composition

The monosaccharide composition was measured using the method described previously [6]. Briefly, 2 mg of polysaccharide sample was hydrolyzed into monosaccharides with 2 N TFA at 120 °C for 1 h. The hydrolyzed sample solution was dried, redissolved in 0.4 mL of deionized water, filtered, and derivatized with 0.5 M 1-phenyl-3-methyl-5-pyrazolone (PMP) at 70 °C for 100 min to obtain the monosaccharide derivatives. The analysis of monosaccharide derivatives was performed by an HPLC system consisting of a UV detector coupled with a Mightysil RP-18 GP column (4.6 × 250 mm, 5 μm; Kanto Chemical, Tokyo, Japan) and a guard column. The derivatized samples and different concentrations (50, 100, 500, 1000, and 2000 μg/mL) of 10 monosaccharide standards (D-mannose, D-ribose, L-rhamnose, D-glucuronic acid, D-galacturonic acid, D-glucose, D-galactose, D-xylose, L-arabinose, and L-fucose) were eluted by a mobile phase consisting of 0.1 M phosphate buffer (pH 6.7) and acetonitrile in a ratio of 83:17 (*v*/*v*) at a flow rate of 1 mL/min. The detection wavelength was 245 nm.

### 2.9. Cell Culture

Human triple-negative breast cancer cell line MDA-MB-231 and human mammary epithelial cell line H184B5F5/M10 were purchased from Bioresource Collection and Research Center (Hsinchu, Taiwan). MDA-MB-231 cells were grown in DMEM, and H184B5F5/M10 cells were cultured in MEM-α. Both media were supplemented with 10% fetal bovine serum, 100 U/mL penicillin, and 100 U/mL streptomycin. Cells were cultured in a 5% CO_2_ atmosphere at 37 °C.

### 2.10. Cytotoxicity Assay

The cytotoxicity of the samples was determined using an MTT assay. MDA-MB-231 cells and H184B5F5/M10 cells (5000 cells/well) were seeded in a 96-well plate and incubated for 24 h, followed by treating with different concentrations (0, 30, 60, 125, 250, 500, and 1000 μg/mL) of F1, F2, and F3 for 24 h. After removing the supernatant, 100 μL of 0.5 mg/mL MTT reagent was added to each well and incubated at 37 °C for 3 h. The supernatant of each well was removed, followed by adding 100 μL of DMSO to solubilize the formazan. The absorbance at 570 nm was measured by a microplate reader (Infinite M200 PRO). Cisplatin (40 μM) was used as a positive control. The IC_50_ value was calculated using SPSS statistic software (Version 28.0, IBM Corp., Armonk, NY, USA).

### 2.11. Cell Cycle Analysis

MDA-MB-231 cells were seeded in 6 cm dishes at a density of 4 × 10^5^ cells/dish. After incubation for 24 h, cells were treated with or without different concentrations (30, 60, and 125 μg/mL) of F2 for 24 h. The floating and attached cells were harvested, fixed in 70% ethanol solution, and kept at −20 °C overnight. After washing with cold PBS, cells were stained with 50 μg/mL propidium iodide (PI) and 200 μg/mL RNase A (dissolved in PBS with 0.1% Triton X-100) at 37 °C for 30 min in the dark. Measurements were performed on 10,000 cells per sample using a flow cytometer (FC500, Beckman Coulter Inc., Brea, CA, USA).

### 2.12. Cell Apoptosis Analysis

MDA-MB-231 cells (4 × 10^5^ cells/dish) were cultured in 6 cm dishes for 24 h and incubated with or without different concentrations (30, 60, and 125 μg/mL) of F2 for 24 h. The floating and attached cells were harvested, washed with cold PBS twice, and stained using Annexin V-FITC apoptosis detection kit (Strong Biotech Corp., New Taipei City, Taiwan) at 37 °C for 15 min in the dark. After labeling, 10,000 cells per sample were analyzed using a flow cytometer (FC500).

### 2.13. Protein Microarray Analysis

Cell cycle-related protein expressions were analyzed using AAH-BLG-CYC-4 Human Cell Cycle Array L1 Glass Slide kit according to the manufacturer’s procedures (RayBiotech, Inc., Norcross, GA, USA). Briefly, MDA-MB-231 cells were collected after treatment with or without 60 μg/mL of F2 for 24 h. Cells were lysed by RIPA lysis buffer containing protease and phosphatase inhibitors, and the cell lysates were kept at −80 °C until analysis. After dialysis, the cell lysates were biotin-labeled and then subjected to secondary dialysis to remove excess reagent. The microarray chips were incubated with biotin-labeled proteins at room temperature for 2 h, followed by incubating with a Cy3-conjugated streptavidin dye for 1 h. The fluorescence intensity of the chip was detected by SureScan Microarray Scanner (Agilent Technologies, Santa Clara, CA, USA).

### 2.14. Statistical Analysis

Data were presented as mean ± standard deviation. Differences between treatment groups were assessed using one-way analysis of variance (ANOVA) and Tukey’s honest significant difference (HSD) test. Student’s *t*-test was used to compare differences between the control and the treatment group. A value of *p* < 0.05 was considered statistically significant.

## 3. Results and Discussion

### 3.1. Yield of SPS, F1, F2, and F3

**Figure 1:**
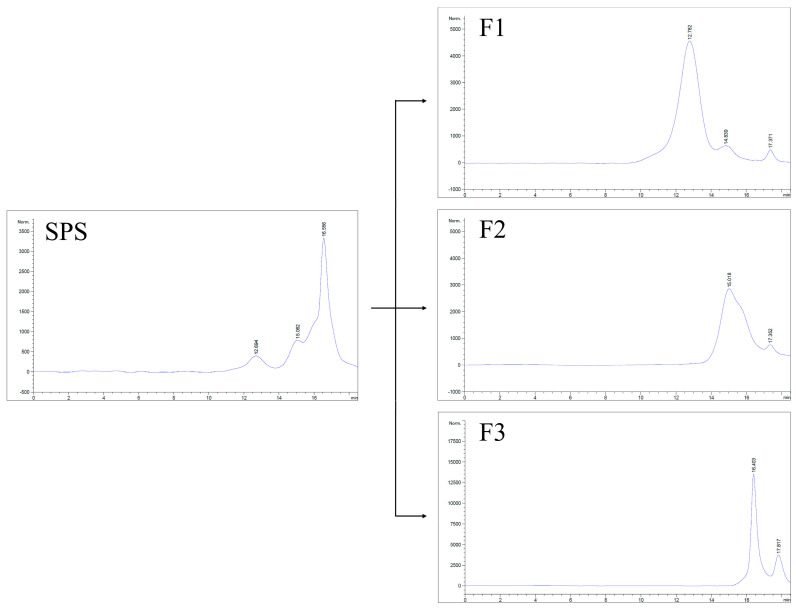
HPGFC profiles of SPS, F1, F2, and F3.

The yield of SPS from *L. sulphureus* fruiting bodies was about 9.75%. The HPGFC profile showed that SPS contained three peaks, which appeared at 12.69, 15.06, and 16.56 min (Figure 1). After isolation and purification, SPS was successfully separated into three components, including the high-molecular-weight SPS (F1), the medium-molecular-weight SPS (F2), and the low-molecular-weight SPS (F3). The yields of F1, F2, and F3 were about 1.6%, 25.5%, and 44.0% of SPS, respectively.

### 3.2. Chemical Composition

Table 1 showed that the total carbohydrate contents of F1, F2, and F3 were 92.95%, 53.86%, and 35.34%, respectively. F1 and F3 contained small proportions of protein (5.12% and 1.55%). Although the polysaccharides were deproteinized by papain during the extraction, a significant amount of protein was found in F2 (15.57%). Previous studies have shown that polysaccharopeptides from *C. versicolor* could resist enzymatic proteolysis [29], indicating that F2 might contain a glycoprotein or protein-bound polysaccharides. The uronic acid contents of F1, F2, and F3 were 12.86%, 4.45%, and 2.70% (Table 1), respectively, suggesting that F1 was an acidic polysaccharide. The sulfate contents were 1.99% in F2 and 2.24% in F3, while there was only 0.24% in F1 (Table 1); this indicates that sulfated polysaccharides were mainly in F2 and F3. The total sugar contents in F2 and F3 were lower than in F1. Although it remains unclear how the sulfate group affects the total sugar contents in various SPS components, the possible reasons for explaining the low total sugar contents in F2 and F3 are as follows: The sulfate group [30] and type of monosaccharide composition [31] have been shown to affect the accurate estimation of the total sugar content in sulfated polysaccharides. Under the phenol sulfuric acid method, the total sugar content was calculated based on the number of sugar molecules present, but samples taken for measurements if they contain high proportions of non-sugar components, such as sulfate and protein contents, may lead to the low total sugar content of the sample. Moreover, due to the negative charge of the sulfate group, sulfated polysaccharides can also easily adsorb the positively charged non-sugar components, such as ash and pigment [11,32]. Given that the sulfate content of F3 was higher than F2, indicating that F3 might have a stronger ability to adsorb positively charged compounds than F2, it therefore resulted in a lower total sugar content.

### 3.3. Monosaccharide Composition

As shown in Table 2, F1 was mainly composed of glucose (84.92%) with some mannose (4.31%), galactose (5.88%), and fucose (4.89%). F2 contained four monosaccharides with similar molar percentages, including mannose (27.70%), glucose (16.65%), galactose (31.58%), and fucose (24.06%). F3 was mainly composed of mannose (38.73%) and glucose (25.07%), and with small amounts of ribose (10.45%), galactose (10.56%), xylose (6.44%), and arabinose (8.76%). These results suggest that F1 was nearly homo-polysaccharides, while F2 and F3 were heteropolysaccharides. Previous studies have shown that the high-molecular-weight polysaccharide component in non-digestible soluble polysaccharides from *X. nigripes* was mainly composed of glucose. In contrast, the low-molecular-weight polysaccharide component was heteropolysaccharides [6]. Similar results were found in the monosaccharide composition of different molecular weight polysaccharide components from *G. frondosa* fruiting bodies [5]. It could be inferred that glucose is the predominant monosaccharide in high-molecular-weight polysaccharide components from mushrooms. In contrast, the low-molecular-weight components mainly comprise different monosaccharide units, forming the heteropolysaccharides.

### 3.4. Molecular Weight

The HPGFC results showed that F1 possessed one major peak at 12.78 min and one minor peak at 14.84 min (Figure 1), which correspond to the molecular weights of 585.9 kDa and 29.3 kDa, respectively. Both F2 and F3 contained only one peak, which appeared at 15.02 and 16.40 min, and their molecular weights were 23.0 kDa and 3.2 kDa, respectively (Figure 1). The peak around 17 min was recognized as a solvent peak (50 mM NaNO_3_ solution containing 0.05% NaN_3_). These results indicate that F1 was a polysaccharide with a high-molecular-weight distribution, and F2 and F3 were polysaccharides with a medium- and a low-molecular-weight distribution, respectively. During the extraction process of SPS, the low-molecular-weight (<10 kDa) substances should be removed by dialysis, however they were detected. Hence, F3, the low-molecular-weight SPS component, could be derived from the breakdown of polysaccharides during the isolation, purification, and drying processes.

Based on the above results, it could be noted that F1, F2, and F3 were three polysaccharides with different physicochemical properties, which may possess various degrees of efficacy in inhibiting breast cancer cell proliferation.

### 3.5. Anti-Proliferative Effects of F1, F2, and F3 on MDA-MB-231 Cells

**Figure 2:**
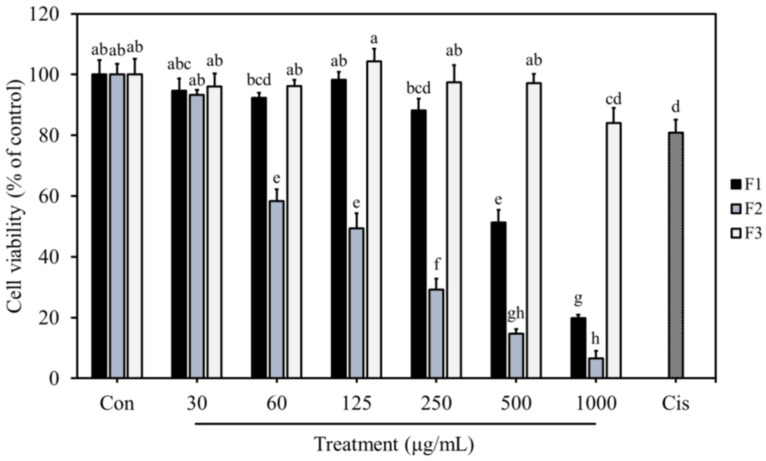
Cytotoxicity effects of different F1, F2, and F3 concentrations on MDA-MB-231 cells. Data are presented as mean ± SD (*n* = 4). The bars with different letters are significantly different (*p* < 0.05), as determined by Tukey’s HSD test. Con: control; Cis: 40 μΜ cisplatin.

**Figure 3:**
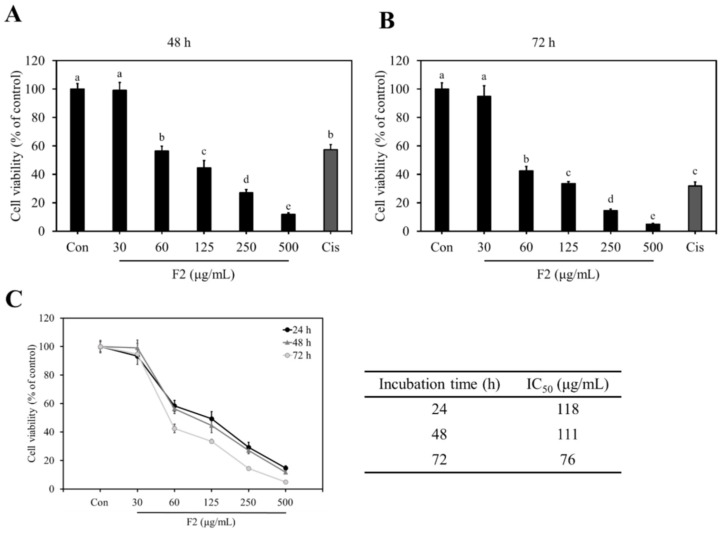
F2 inhibited the viability of MDA-MB-231 cells. Cells were treated with different concentrations of F2 for (**A**) 48 h and (**B**) 72 h. (**C**) The cell viability and IC_50_ values of F2 at various incubation times. Data are presented as mean ± SD (*n* = 4). The bars with different letters are significantly different (*p* < 0.05), as determined by Tukey’s HSD test. Con: control; Cis: 40 μΜ cisplatin.

The cytotoxic effects of F1, F2, and F3 on MDA-MB-231 cells were examined by MTT assay. The results showed that breast cancer cell proliferation was significantly inhibited after treating with F1 and F2 for 24 h (Figure 2). At 60 μg/mL, F2 markedly reduced the cell proliferation by 41.7%, which was higher than F1 (7.7%) and F3 (3.8%) (Figure 2). After increasing the treatment concentration to 500 μg/mL, F1 significantly inhibited the cell proliferation with an inhibition rate of 48.7% (Figure 2). However, F3 did not exhibit a cytotoxic effect on breast cancer cells until increasing the treatment concentration to 1000 μg/mL. Cisplatin, a clinical anti-breast cancer drug, was used as a positive control in this study. Under 40 μM of cisplatin, the viability of MDA-MB-231 cells was reduced by 19.1% (Figure 2). The IC_50_ values of F1, F2, and F3 were 537 μg/mL, 118 μg/mL, and above 1000 μg/mL, respectively; this suggests that F2 possessed better anti-proliferative activity against MDA-MB-231 cells than F1 and F3. Furthermore, F2 also significantly inhibited the cell viability after 48 and 72 h of treatments, with an IC_50_ value of 111 and 76 μg/mL, respectively (Figure 3).

It has been noted that the high degree of sulfation could improve the solubility and increase the negative charges of polysaccharides, leading to enhanced anti-cancer and immunomodulatory activity [4]. A previous study demonstrated that sulfated polysaccharides from Radix hedysari displayed better anti-cancer activity than native polysaccharides [33]. In this study, the sulfate content of F3 was higher than F2, whereas the anti-proliferative activity of F3 was weaker than F2. F1 contained a very low amount of sulfate, but its cytotoxic effect on MDA-MB-231 cells was better than F3. It is possible that in addition to the sulfate content, the molecular weight may have also contributed to the differences in the anti-cancer activity between F1 and F3, as supported by a previous study, indicating that acidic polysaccharides with the highest molecular weight from *Sanhuangporus vaninii* exhibited the most potent anti-cancer activity against lung cancer cells [34]. This observation suggests that molecular weight is also an important factor affecting the bioactivity of sulfated polysaccharides.

Uronic acid is a common component in polysaccharides. Due to the presence of carboxyl functional groups, uronic acid-containing polysaccharides are negatively charged polymers, which are supposed to have more potent bioactivity than neutral polysaccharides [35,36]. Although carboxyl and sulfate groups are both negatively charged functional groups, they have different charges, which may lead to different bioactivity [37]. Recent studies have pointed out that sulfated glycan containing no uronic acid and a high degree of sulfation (DS) showed better anti-viral activity than sulfated polysaccharides composed of high uronic acid and lower DS; this observation suggests that the sulfate group plays a vital role in bioactivity rather than the carboxyl group [38]. For this reason, although F1 was a high-molecular-weight SPS containing high uronic acid, its anti-proliferative activity was not as potent as F2, which had a higher sulfate content. In addition, F2 contained the highest amount of protein among the three SPS components. Protein and peptide units were reported to make polysaccharides more flexible and easily recognized by cell membrane receptors, consequently leading to better bioactivity [39].

Monosaccharides are the building blocks of polysaccharides, which affect their structure, bioactivity, and effectiveness. F1, F2, and F3 were sulfated polysaccharides with significant differences in their monosaccharide composition and molar ratio of monosaccharides. F2 possessed the most potent anti-breast cancer activity; it had higher galactose and fucose proportions compared to F1 and F3; these results correspond to a previous study indicating that galactose and fucose were the monosaccharides, which had the most significant impact on the bioactivities of polysaccharides [40]. In addition, studies have also demonstrated that terminal fucosylation of mushroom polysaccharides played an important role in the anti-tumor and immunomodulatory activities [41,42]. These results indicate that the galactose and fucose units might contribute to the enhanced anti-breast cancer activity of F2.

The bioactivities of polysaccharides are contributed to by their complex and diversified physicochemical properties. Observed bioactivities in cells and organisms are the results of the interaction between these characteristic parameters. Under different conditions, the main factors affecting bioactivity might be different. Based on the above results, F1, F2, and F3 were shown to have varying degrees of cytotoxic effects on MDA-MB-231 cells due to their chemical structural differences. The molecular weight, monosaccharide composition, and protein content might be the key factors affecting the anti-breast cancer activity of sulfated polysaccharides when MDA-MB-231 cells were used as a model cell line. In addition, previous studies have shown that different polysaccharide components with distinct physicochemical properties exhibited a synergistic effect [6]. It is also possible that F1, F2, and F3 may interact to produce an enhanced bioactivity. Therefore, further studies on the interaction of these three SPS components are also necessary.

### 3.6. Effects of F1, F2, and F3 on Normal Cell Proliferation

**Figure 4:**
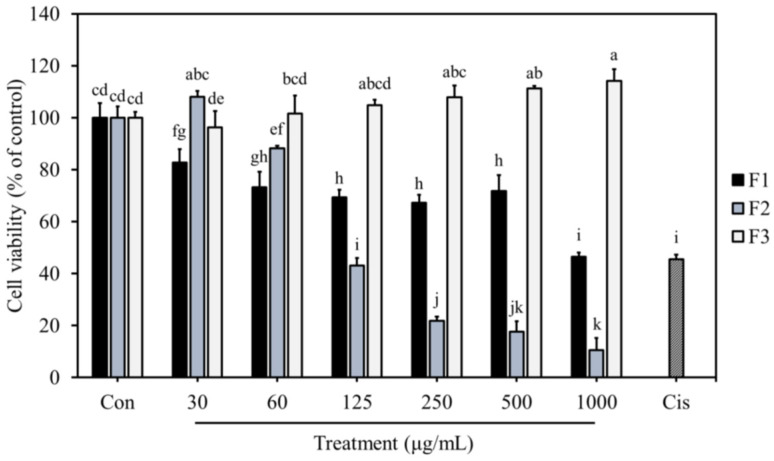
Effects of F1, F2, and F3 on H184B5F5/M10 cell proliferation. Cells were treated with different F1, F2, and F3 concentrations for 24 h. Data are presented as mean ± SD (*n* = 4). The bars with different letters are significantly different (*p* < 0.05), as determined by Tukey’s HSD test. Con: control; Cis: 40 μΜ cisplatin.

Since chemotherapy drugs may damage both cancer and normal cells, selective cytotoxicity is recognized as an important criterion for developing novel anti-cancer drugs [43]. As shown in Figure 4, F1 at a low concentration (30 μg/mL) already possessed cytotoxicity to H184B5F5/M10 cells, the normal mammary cell line. The viabilities of normal mammary cells were about 70–80% after treatment with 30–500 μg/mL of F1. This suggests that F1 might cause slight cytotoxicity to normal mammary cells. After treatment with 30 μg/mL of F2, the cell viability of H184B5F5/M10 cells was not significantly affected compared to the control group; however, when the concentration was raised to 60 μg/mL, the cell viability decreased to 88.3%. At the same concentration (60 μg/mL), F2 showed much more potent cytotoxicity on MDA-MB-231 breast cancer cells. It caused the cell viability to be reduced to 58.3%, indicating that F2 exhibited selective cytotoxicity to breast cancer cells. At concentrations as high as 1000 μg/mL, F3 showed no or a weak cytotoxic effect on H184B5F5/M10 cells and MDA-MB-231 cells. These results conclude that F2 showed selective cytotoxicity to MDA-MB-231 cells at the low concentration range, whereas F1 and F3 were cytotoxic to normal cells and/or weak in inhibiting breast cancer cell proliferation.

Plant polysaccharides isolated from the genus *Astragalus*, *Ginseng*, and *Schisandra* were found to possess selective cytotoxicity toward cancer cells by triggering cancer cell apoptosis [44]. Other studies have also reported that polysaccharides from *Porphyra haitanensis* exhibited selective anti-colon cancer activity via cell cycle arrest and apoptosis [45]. The selective anti-cancer activity induced by chitin was mainly due to the interaction with charged components on the surface of cancer cells, leading to the disruption of cell membranes [46]. The selective cytotoxicity of polysaccharides and SPS could be the major factor contributing to their increasing investigations for potential use as anti-cancer agents, which are safer than conventional chemotherapy drugs.

### 3.7. F2 Induced Cell Cycle Arrest but Not Apoptosis

**Figure 5:**
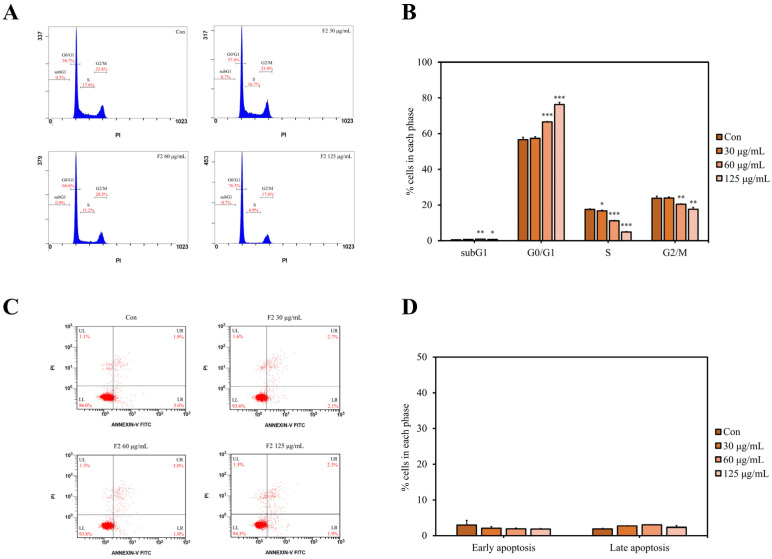
F2 induced cell cycle arrest but not apoptosis in MDA-MB-231 cells. Cells were treated with or without different concentrations of F2 for 24 h. (**A**) Representative flow cytometric graphs of cells. (**B**) The percentages of cells in each cell cycle phase. (**C**) Representative dot plots of PI and Annexing V staining cells. (**D**) The percentages of early apoptotic cells and late apoptotic cells. Data are presented as mean ± SD (*n* = 3). Significant differences compared to the control are indicated by Student’s *t*-test (* *p* < 0.05; ** *p* < 0.01; *** *p* < 0.001). Con: control.

Cell cycle arrest and apoptosis are the two major pathways controlling cell survival and death. They are also two well-known strategies to terminate the uncontrolled proliferation in cancer cells [47]. As shown in Figure 5A,B, after treating with F2 for 24 h, the percentage of cells at the G0/G1 phase was significantly increased, whereas those at the S and G2/M phases were markedly reduced. However, the percentages of cells in the early and late apoptosis stages did not change substantially after treatment with F2 for 24 h (Figure 5C,D). These results indicate that F2 suppressed breast cancer cell proliferation mainly by blocking the cell cycle at the G0/G1 phase but not triggering cell apoptosis.

Studies have shown that mushroom polysaccharides can reduce cancer cell proliferation via cell cycle arrest [48,49,50]. Water-soluble polysaccharides from *G. lucidum* were reported to inhibit lung cancer cell proliferation through MAPK/ERK, PI3K/Akt, Smad, and FAK signaling pathways, leading to G1 phase arrest but not apoptosis [51]. These studies indicate cell cycle arrest is the major anti-cancer pathway triggered by the SPS of *L. sulphureus*, especially the F2 component.

### 3.8. Mechanistic Effects of F2 on Proteins Related to the Cell Cycle Arrest Pathway

**Figure 6:**
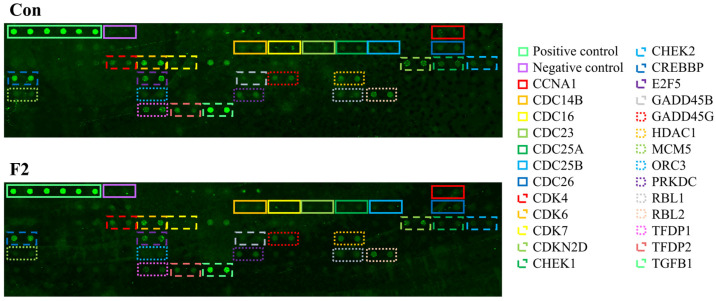
Representative protein chips from Human Cell Cycle Antibody Array analysis. MDA-MB-231 cells were treated with or without 60 μg/mL of F2 for 24 h. Con: control.

To decipher the possible pathways involved in F2-induced cell cycle arrest, protein microarray analysis was used to detect the differentially expressed cell cycle-related proteins between the control and F2 groups. As shown in Figure 6, the intensity of the fluorescence signal on the chip represented the degree of protein expression. The bright green signals on the upper left side are positive controls, which can be used to normalize the results between arrays. Compared to the control group (Con), 26 proteins were noted to change after treatment with F2 (Table 3 and Figure 6). F2 caused the down-regulation of CCNA1, CDC14B, CDC16, CDC23, CDC25A, CDC25B, CDC26, CDK4, CDK6, CDK7, CHEK-1, CHEK-2, CREBBP, E2F5, GADD45B, GADD45G, HDAC1, MCM5, ORC3, PRKDC, RBL1, RBL2, TFDP1, TFDP2, and TGFB1 protein expressions, while it enhanced the expression of the CDKN2D protein in MDA-MB-231 cells.

The results demonstrate that F2 mainly targeted the cell cycle checkpoint and cell division-related proteins in MDA-MB-231 cells. Proteins involved in the G1 phase progression and G1/S transition, such as CDK4, CDK6, CDK7, CDKN2D, E2F5, RBL1, RBL2, TFDP1, TFDP2, CDC25A, and CDKN2D, were affected by F2, which is consistent with the expected G0/G1 phase arrest. The cell cycle comprises four stages: G1, S, G2, and M phases. In addition to these four phases, there is a quiescent stage called the G0 phase, which is out of the cell cycle. Once DNA damage occurs, checkpoints provide an opportunity to halt cell cycle progression and prevent the replication of damaged DNA [52]. Cell cycle progression is tightly regulated by cyclin-dependent kinases (CDKs), and their regulatory protein subunit is called cyclins. The CDK is inactive but activated when forming a cyclin–CDK complex with cyclins [53]. These complexes can drive the cell cycle, promoting DNA replication and cell division. When CDK4 and CDK6 form complexes with cyclin D, they phosphorylate and inactivate the retinoblastoma protein (Rb1), leading to the release of the E2F transcription factor, which in turn allows the cell cycle to progress from the G1 to S phase [54,55]. Activated CDK7 phosphorylates CDK1, CDK2, CDK4, and CDK6, resulting in the activation of CDKs and cell cycle progression [56]. Studies have shown that the down-regulation of CDC25A, a protein phosphatase required for cell cycle progression, may block G1/S transition [57]. Up-regulation of p19 has been shown to inhibit the formation of the cyclin D–CDK4/6 complex, leading to G0/G1 phase arrest [58]. CDC14B is a phosphatase that can dephosphorylate CDK substrates and is essential for the regulation of mitosis and DNA replication [59]. CDC16, CDC23, and CDC26 are subunits of the anaphase-promoting complex/cyclosome, which play an important role in the transition from the metaphase to the anaphase [60]. CREBBP, HDAC1, MCM5, ORC3, and PRKDC are proteins involved in DNA replication. CREBBP is a histone lysine acetyltransferase that can reduce the affinity between DNA and histones, thereby promoting DNA replication and gene transcription. Conversely, HDAC1 is a histone deacetylase, which has the opposite function of CREBBP [61]. Once the cell cycle was blocked at the G0/G1 phase by F2, the progression of the S and M phases was also retarded, leading to a decrease in DNA replication and mitosis. This could be explained by the responses of proteins related to DNA replication, spindle formation, and cell division, such as the expression of CDC14B, CDC16, CDC23, CDC25A, CDC25B, CDC26, CREBBP, HDAC1, MCM5, ORC3, and PRKDC proteins after treatment with F2.

TGFB1, which encodes TGFβ1 (transforming growth factor β1), is a multi-functional cytokine regulating cell proliferation, differentiation, growth, and extracellular matrix synthesis. In the cell cycle, TGFβ1 is demonstrated to inhibit the phosphorylation of Rb1 and down-regulate c-myc transcription, subsequently causing cell cycle arrests at the G1 phase [62]. The results showed that the expression of TGFβ1 in MDA-MB-231 cells decreased after exposure to F2, suggesting that although F2 could regulate the TGFβ signaling pathway, the decrease in TGFβ1 might not be directly involved in F2-induced cell cycle arrest. In addition to the cell cycle, other studies have pointed out that the TGFβ signaling pathway also mediates cell migration and invasion by promoting epithelial–mesenchymal transformation, an important cancer metastasis process [63].

## 4. Conclusions

This study demonstrated the differences in the physicochemical and anti-breast cancer properties of three SPS components, F1, F2, and F3, from *L. sulphureus* fruiting bodies. F1, the high-molecular-weight SPS component (585.9 and 29.3 kDa), was mainly composed of glucose and had the highest uronic acid content (12.86%). F2, the medium-molecular-weight SPS component (23.0 kDa), was composed of mannose, glucose, galactose, and fucose, with the highest protein content (15.57%) and a significant amount of sulfate (1.99%). The low-molecular-weight SPS component (3.2 kDa) named F3 comprised various monosaccharide units and had the highest sulfate content (2.24%). Among the three components, F2 showed the most potent cytotoxicity against MDA-MB-231 cells, suggesting that the molecular weight, monosaccharide composition, and protein content may be the key factors affecting the anti-breast cancer activity of sulfated polysaccharides. F2 exhibited anti-proliferative activity by arresting the cell cycle at the G0/G1 phase but not triggering the cell apoptosis. Moreover, F2 also had selective cytotoxicity against breast cancer cells. The protein microarray results indicated that after treatment with F2, the expressions of 26 proteins involved in G0/G1 phase progression, cell cycle checkpoints, and DNA replication were changed compared to the control. In addition, F2 could regulate the TGFβ signaling pathway in MDA-MB-231 cells. These results conclude that F2, the medium-molecular-weight SPS of *L. sulphureus*, exerts selective anti-breast cancer activity and could be considered as an adjuvant or a potential drug candidate.

## Figures and Tables

**Table 1:** Chemical composition of F1, F2, and F3.

Sample	Chemical Composition (%)			
	Total Sugar	Total Protein	Uronic Acid	Sulfate
F1	92.95 ± 0.10	5.12 ± 0.26	12.86 ± 2.10	0.24 ± 0.02
F2	53.86 ± 3.62	15.57 ± 2.44	4.45 ± 0.42	1.99 ± 0.07
F3	35.34 ± 9.00	1.55 ± 0.34	2.70 ± 0.89	2.24 ± 0.02

Data are presented as mean ± SD (*n* = 3).

**Table 2:** Monosaccharide composition of F1, F2, and F3.

Sample	Monosaccharide Composition (Molar %)						
	Man	Rib	Glc	Gal	Xyl	Ara	Fuc
F1	4.31 ± 0.02	ND	84.92 ± 0.07	5.88 ± 0.03	ND	ND	4.89 ± 0.01
F2	27.70 ± 3.61	ND	16.65 ± 2.22	31.58 ± 3.58	ND	ND	24.06 ± 2.43
F3	38.73 ± 4.33	10.45 ± 1.26	25.07 ± 7.79	10.56 ± 0.56	6.44 ± 0.59	8.76 ± 1.09	ND

Data are presented as mean ± SD (*n* = 3); Man: mannose; Rib: ribose; Glc: glucose; Gal: galactose; Xyl: xylose; Ara: arabinose; Fuc: fucose; ND: non-detected.

**Table 3:** Cell cycle-related protein expressions in MDA-MB-231 cells.

Target Gene Name	Target Protein Name	Fluorescence	
		Con	F2
POS-Ave (positive control)		26,477	26,477
Neg (negative control)		775	642
CCNA1	Cyclin A1	1186	863
CDC14B	Dual specificity protein phosphatase	1504	1095
CDC16	Cell division cycle protein 16 homolog	2222	1225
CDC23	Cell division cycle protein 23 homolog	1135	651
CDC25A	Cell division cycle protein 25A	1403	736
CDC25B	Cell division cycle protein 25B	1003	823
CDC26	Cell division cycle protein 26 homolog	1424	1183
CDK4	Cyclin-dependent kinase 4	2882	2137
CDK6	Cyclin-dependent kinase 6	11,611	8464
CDK7	Cyclin-dependent kinase 7	1261	362
CDKN2D	Cyclin-dependent kinase 4 inhibitor p19	4099	5613
CHEK1	Cell cycle checkpoint kinase 1	2247	1004
CHEK2	Cell cycle checkpoint kinase 2	1360	898
CREBBP	Histone lysine acetyltransferase	12,918	7627
E2F5	Transcription factor E2F5	7486	4780
GADD45B	Myeloid differentiation primary response protein	1352	909
GADD45G	DNA damage-inducible transcript 2 protein	2843	2397
HDAC1	Histone deacetylase 1	3731	3367
MCM5	DNA replication licensing factor MCM5	1675	1043
ORC3	Origin recognition complex subunit 3	1413	709
PRKDC	DNA-dependent protein kinase catalytic subunit	2996	2602
RBL1	Retinoblastoma-like protein 1	2010	1662
RBL2	Retinoblastoma-like protein 2	4160	2878
TFDP1	Transcription factor Dp-1	5082	1639
TFDP2	Transcription factor Dp-2	5290	2268
TGFB1	Transforming growth factor β1	32,173	19,476

Con: control. Cells were treated with or without 60 μg/mL of F2 for 24 h and analyzed by Human Cell Cycle Array.

## Data Availability

Data will be made available on request.
